# Commentary: Dynamic Upper Eyelid Reconstruction for Total Periorbital Soft Tissue Loss

**DOI:** 10.1055/s-0043-1777240

**Published:** 2024-02-22

**Authors:** Madhubari Vathulya

**Affiliations:** 1 Department of Burns and Plastic Surgery, All India Institute of Medical Sciences, Rishikesh, Uttarakhand, India

Dynamic Upper Eyelid Reconstruction for Total Periorbital Soft Tissue Loss

Total eyelid defect comprises full-thickness loss of both upper and lower eyelids in a patient. It is a rare and devastating condition with serious implications related to vision, which mandates early and functional reconstruction when associated with intact globe. The primary goal is to give a stable coverage for orbital protection but at the same time provide a functional reconstruction of the defect, to allow for adequate mobility of the eyelids so that the patient's vision is restored to normal with minimal disability. When the defect is massive, and in the absence of loco-regional flaps, microvascular tissue transfer is needed. In this report we describe a radial-artery-based microvascular tissue transfer with a unique innovation utilizing the contralateral frontalis muscle to reconstruct a case of unilateral total upper and lower eyelid loss.



